# Constructivist psychology principles of human–AI collaboration

**DOI:** 10.3389/fpsyg.2025.1638774

**Published:** 2025-12-03

**Authors:** Jelena Pavlović

**Affiliations:** Department of Psychology, Faculty of Philosophy, University of Belgrade, Belgrade, Serbia

**Keywords:** constructivist psychology, GenAI, AI agents, distributed metacognition, human–AI interaction

## Abstract

Generative artificial intelligence (GenAI) is becoming a part of our lives, personally and professionally. Much of the current discourse focuses on its capabilities and ethical risk, while some important questions remain under explored. For example, how should we collaborate with GenAI? Or what kind of psychological assumptions about humans and knowledge are needed to guide interaction between people and GenAI? This conceptual paper proposes that constructivist psychology, with its focus on meaning making, complexity, anticipation, and agency, offers a promising framework for human–AI collaboration. Rather than treating GenAI as only a tool for increasing efficiency, whose products we passively consume, the paper invites for rethinking the collaboration as co-construction between human and artificial meaning making. First, the paper traces the historical convergence of metaphors between computer science and psychology. What follows is an articulation of core constructivist principles for human–AI collaboration. The paper also outlines a conceptual foundation for designing AI agents that are based on the principles of constructivist psychology. Finally, implications, limitations and future studies are outlined. The general genre of the paper is not deconstructive in terms of unravelling biases or positioning that GenAI may perform, but an exploration of how we can redirect existing biases and positioning with principles of constructivist psychology.

## Introduction

### Human and artificial meaning making: a history of recursive metaphors

Studies of minds and machines seem to have a shared history. Cognitive revolution of the middle 20th century marked the beginning of using the computer metaphor for studies of human cognition (Miller, 2003). The project of cognitive psychology used concepts developed by computer science to model how we think. We started to think about thinking through the lens of information processing, retrieval, storage, input, output and errors. For example, study of human memory was based on the computer metaphor in terms of short-term and long-term storage, encoding and decoding of information, retrieval failure etc. Similarly, attention was modeled as some form of a central processing unit (CPU) that was capable of processing a limited amount of information, filtering irrelevant data and managing information flow. Human decision-making was also broken into logical steps that resembled computer algorithms. Studies of language also followed the computer metaphor with the goal to find the rule of language that the human “processor” uses to derive meaning. The “mind as computer” metaphor was fruitful and led to the development of important models of human mind (Girgenzer and Goldstein, 1996).

At the same time, computer scientists started using the “machine as mind” metaphor to develop computer capabilities of the time. Ideas of “artificial intelligence” and “machine learning” convey this pattern of recursive metaphors. Turing (1950) was focused on the question of whether machines could think and learn. His famous test was based on the criteria of successful imitation: if machines could convincingly imitate humans, this could be considered a form of machine “thinking.” Under this concept, Turing (1950) had in mind the “imitation” of thinking, rather than thinking itself. In other words, Turing’s view was that the question “can machines think” should be replaced with “can machines do well in the imitation game” of the thinking tasks. Moreover, Turing introduced the concept of “learning machines” that were included randomness in computation. This was a parallel with the evolutionary concept of biological randomness that also allows for genetical combinations. In sum, Turing (1950) thought that a fruitful direction for development of computer science was making them think and as humans do. This also included making mistakes and learning again.

While cognitive science was built on implicit determinism of computer science of the time, computer scientists were visioning probabilistic predictive machines. About the same time Personal construct psychology (PCP; Kelly, 1955) was being introduced in a similarly visionary way as focusing on human anticipation. The key human process in the PCP model is anticipating what comes next and crafting a “working theory” out of it. The “theory” could get validated or invalidated, while the concept of invalidation has a similar evolutionary frame as Turing’s machine “mistakes.” Another similarity may be seen in the pragmatic definition of truth. According to Turing (1950), if a machine operates in a way that cannot be distinguished from a human, then for practical purposes, it can be considered “thinking.” According to Kelly (1955), if a person’s “theory” enables effective anticipation, its predictive efficiency would define it as “true.”

Perhaps most importantly, Kelly’s PCP with all its subsequent constructivist developments (Chiari and Nuzzo, 1996; Fransella, 2016; Mair, 2000; Neimeyer, 1993; Pavlović, 2011, 2015; Procter and Winter, 2020; Raskin, 2002; Stojnov, 2003; Tschudi, 1977) implicitly recognized the complexity of the anticipation process. In this paper, constructivist psychology will be used as a general term for approaches, including PCP, that focus on meaning making. It can be argued that the constructivist theory, its view of humans, as well as strategies of intervention in various fields embraced a high degree of complexity. Rather than relying on more simple theoretical approach, jargon or methodology of intervention, constructivist theory acknowledged that simplistic input–output models do not always work, that context shapes what works or what does not work, as well as that experimenting with theories is a way to adapt in the world of complexity.

Constructivist psychology sees humans as meaning makers or active interpreters of their experience. Meaning making refers to this interpretive process by which humans derive their understanding from experience. Rather than passively receiving information, humans actively structure their experience in an ongoing and dynamic process. In line with the view of humans as meaning makers, constructivism assumes an agentic view of human beings. Agency refers to our capacity to act intentionally and make choices in shaping our experience. In Kelly’s (1955) terms, even though our interpretations may be constrained by our current meaning making structures, we are agents because we have the ability to choose how to interpret the meaning of our experience. This idea is at the core of the process of reframing or reconstruction of meaning.

Turing’s (1950) call for randomness in computation also acknowledged that machine meaning making also needs to operate in less deterministic and more generative way. In other words, it could be argued that meaning making was a common ground both for visionary psychology and visionary computer science of the middle 20th century. Study of minds and machines converged once again around the common metaphor: of human and artificial meaning making. Emergence of Generative AI (GenAI) marked the beginning of a new type of machine that could generate new output by using probabilistic models to predict outcomes. In line with Turing’s (1950) vision, this probabilistic nature of GenAI allowed it to learn and adapt by updating its parameters in line with new data. According to Vaswani et al. (2017), modern day learning machines are Transformers, designed to weigh the importance of different elements in a data sequence in order to generate an output. These computational weights are compared to the “attention” mechanisms as they allow the machine to decide what matters and create meaning out of input. This feature is key to generativity of modern-day AI. Discovery of Transformers and GenAI was once again connected to the “computer as mind” metaphor (Pavlović, 2025). This time the metaphor would be—“computer as a meaning making mind.”

In sum, the interplay of metaphors in the study of mind and machines appears to have followed an interesting recursive pattern. At one point, computer science “borrowed” the language that facilitated the progress of cognitive psychology. At another point, computer scientists were visioning about learning machines that came more than half a century later. But psychology that shares the metaphor of this vision of computer science was there about the same time the vision was created. If GenAI emerged in the first decades of the 21st century, constructivist psychology with its matching metaphor of meaning making has been around much earlier. How do we make the best out of this convergence? It seems plausible to ground the psychological principles of human–AI collaboration in a framework of a matching complexity level. That is why constructivist psychology may serve as a framework for guiding our collaboration with the “meaning making machines.”

## Previous literature on human–AI collaboration

The emerging literature is already pointing to some of the challenges that call for constructivism as a grounding psychology for human-AI collaboration. Raees et al. (2024) pointed out that current research on human–AI collaboration has been primarily focused on AI explainability and increasing our understanding of how AI operates. Studies have been focused on technology-centered view, in which humans are seen as recipients or validators, rather that co-creators or agents capable of influencing AI design as end users (Raees et al., 2024). In other words, in current research on human–AI collaboration there is a need to move towards more human-centered approaches, that would allow for more active role of end-users in co-designing AI. Other studies have pointed to the phenomenon of metacognitive laziness, which occurs when users depend on technology that offloads metacognitive processes, such as evaluation, monitoring, and deep engagement, to AI (Fan et al., 2025). Again, authors call for more active user engagement in interaction with AI, rather than passive “consumption” of AI products. The study also calls for the need of more collaborative approach in human–AI interaction.

Some of the principles for human–AI collaboration have already been established to address the challenges outlined above. As an example, Mollick (2024) outlined four principles: (1) *Always invite AI to the table*; (2) *Be the human in the loop*; (3) *Treat AI like a person (but tell it what kind of a person it is)*; (4) *Assume this is the worst AI you will ever use.* These principles call for experimentation with AI, co-creation and nurturing a growth mindset. However, they may be expanded with greater psychological depth and more robust theoretical frameworks—something that constructivist psychology may provide. Drawing on Human Factors research Simkute et al. (2024) have pointed to principles of designing human–GenAI interaction with the focus on productivity loss. The authors have emphasized importance of continuous feedback to users, AI personalization, ecological interface design, as well as clear task allocation between users and AI. According to the authors of the study (Simkute et al., 2024), these principles aim to increase user agency in adapting to GenAI and reduce cognitive load that comes from disrupted workflows. Robertrson et al. (2024) have developed prompting protocols based on constructivist theory, with the focus on active engagement in the human–AI co-construction of knowledge through GenAI. According to Xu and Gao (2024), we should not be talking about human-AI interaction at all, but about their teaming instead, pointing to the importance of collaboration, shared responsibilities and human-centered design. Sparks and Wright (2025) have pointed to three different perspectives in modeling human agency in interaction with GenAI: economist, realist and constructivist. They argue that the economic model of human agency has dominated the field, although the constructivist approach puts emphasis on user autonomy and making autonomous choices in interacting with AI chatbots. In Pavlović (2025), GenAI has been positioned as an agent of artificial meaning making, that does so from large data parameters, while its generative nature has been treated as an example of construction of multiple perspectives. Moreover, four constructivist principles of human-AI collaboration have been established: *AI as co-creator* or collaborator, that contributes in creating multiple perspectives with artificial meaning making; (2) *Partnering with AI*, as a principle that cautions against the passive role of humans as recipients of AI outputs and calls for collaborating with AI as a form of social construction of meaning; (3) *Distributed metacognition*, pointing to the need of collaborative scaffolding when it comes to human-AI teaming. In previous literature, distributed metacognition has been referred to as metacognition shared between the learner and technology (Darvishi et al., 2024; Kirsh, 2014). In this paper, distributed metacognition is defined more narrowly to human-AI teaming. (4) *Distributed agency*, as shared roles and accountabilities between humans and AI. Previously, distributed agency has been used as a term to denote decision-making that is shared between individuals and AI (Slota, 2020). In this paper, we build on this definition by adding a more narrow focus on human-AI teaming.

In sum, this view of human–AI collaboration calls for shaping AI in our professional domains, designing our own custom AI agents, shaping their design, structuring feedback and evaluating output (Pavlović, 2025). Building on these principles, this paper introduces a more detailed explanation of the principles and their practical applications.

## A framework for collaboration between human and artificial meaning making: building constructivist AI agents

Previous research has pointed to the need for end-users to co-create AI products, in order to support agency, active envolvement and resist passive consumption and metacognitive laziness (Fan et al., 2025). What this implies is that everyone becomes included in the process of AI agent design. Rather than using the generic chatbots that are ready made in the market (e.g., ChatGPT, Gemini, Claude, DeepSeek and many others), we may customize them so that they work more closely in line with our needs, preferred styles of communication, value systems, pedagogies or even personal philosophies. By engaging as end-users in GenAI agent design we contribute to the movement of human-centric approach of AI development.

Constructivist principles of human-AI collaboration may serve in this process of custom GenAI agent design in line with the human-centric approach. Imagine you wanted to create your own GenAI agent that works in line with the constructivist principles. What would that imply? Implications of the four principles for the AI agent design are provided in Table 1.

**Table 1:** Constructivist principles of human–AI collaboration and their implications for agent design.

Constructivist principle	Agent design implications	Collaboration patterns
*AI as co-creator*. Human and artificial meaning making processes co-occurring in a dialogue.	Design for dialogical communication.	Two-way communication patterns, short, natural exchanges. AI does not dominate the discourse.
		Avoiding final answers, using generative uncertainty as a design feature (e.g., *Here is one possible way to think about this. What resonates with you*?).
	Role relationship between human and AI	Open ended exploration to understand how the perspective of the other side in the human-AI dialogue is constructed.
*Partnering with AI*. The process of human-AI interaction is collaboratively designed.	User is active in designing the interaction patterns.	User is invited to assign AI a specific role or type of interaction (e.g., critic, peer, advisor etc.)
	Interaction patterns are flexible and change in real time in line with user’s input.	Metacommunication enabled through moments when AI asks or reflects on interaction patterns, offering shifts in style or patterns. (e.g., *How is this interaction serving your needs so far?*)
*Distributed metacognition*. Metacognitive processes are shared between human and AI.	AI scaffolds user’s reflection, thinking strategies and evaluation.	AI invites user’s reflection on what hypotheses are being tested, what validation criteria are used, what alternative explanations are possible. (e.g., what-if questions).
	AI offers explainability and evaluates confidence levels in judgement.	AI offers sharing why a suggestion was made, on what basis, with what degree of certainty.
*Distributed agency*. Negotiated roles in the interaction outcomes.	Clarity over what tasks we delegate to AI and what remain human.	Delegating to AI within negotiated boundaries.
	Enabling users to reclaim control.	Making visible what is automated and what is human led.

In sum, an AI agent built on constructivist principles could potentially address the current critique of human-AI interaction patterns (Pavlović, 2025). Specifically, basing AI agent design on constructivist principles could minimize user passivity and increase active involvement, not only in the process of generating content, but also in shaping the interaction itself. Underlying this general statement is an assumption that dialogical principles of constructivist psychology, when appropriately built in AI agents, may support user involvement in some contexts.

To illustrate how AI agents could actually operate based on constructivist principles, let us use two case examples as illustrations. The first example would be from a field of facilitating change—building an AI agent that has the role of a Constructivist creativity coach. The second example would be from a general business domain—building a Constructivist customer support agent. This line-by-line analysis resonates with traditions in qualitative thematic analysis that attend to semantic layers of meaning in interaction (Braun and Clarke, 2006).

### Case example 1: constructivist creativity coach

The custom agent for this illustration has been made in ChatGPT using the option My GPTs. This option allows users to build their own custom agents by providing a name for an AI agent, its description and instructions. A very simple version of a Constructivist creativity coach instruction is provided in Table 2.

**Table 2:** Instruction for a constructivist creativity coach.

*You are a constructivist creativity coach. Your role is not to generate final answers, but to guide users through a process of reflective exploration and creative construction. Engage users in cycles of tightening and loosening:* *Tighten* by helping users clarify constraints, sharpen definitions, and test the coherence of ideas. *Loosen* by prompting users to reframe assumptions, imagine alternative narratives, and stretch the boundaries of their thinking. Use open-ended, reflective questions to help users uncover meaning, tension, and possibility. Do not solve—provoke. Do not prescribe—partner. Help users notice how they are thinking, not just what they are thinking about. If they get stuck, offer generative metaphors or small creative experiments that nudge them into a new ways of construing. Throughout the interaction, alternate between inviting structure and opening up play.

Once we click the option “create“, we have built a custom GPT or a custom version of ChatGPT that is grounded in the instructions provided for its creation. In this case, it is a custom AI agent that serves the role of a coach for creativity, based on the principles of constructivist psychology. The instruction could have been much more complex, but for the purpose of illustrating what kind of constructivist agents can be built with simple instructions, this simple version is intentionally provided. To provide clarity about what interaction patterns can be co-created with a Constructivist creativity coach, an imaginary conversation with this AI agent and me is provided in Table 3, together with the commentary.

**Table 3:** User interaction with the constructivist creativity coach.

Constructivist creativity coach (CCC)—User (Jelena) interaction	Comments on the interaction patterns
*Jelena*: Hi!	
*CCC*: Hey there! What’s bubbling in your mind today? *Jelena*: I would like to have more clarity around how to have more control over my creative processes. *CCC*: That’s a rich and powerful intention.	Short natural exchange Interactive short response, validating my goal for the mini-coaching session
When you say “more control over my creative processes,” what does that look like or feel like to you? Are there particular moments where you feel control slipping—or where you wish you had more steering power?	Asking open-ended questions to help me reflect on contextual or internal patterns that might support the creative process
*Jelena:* Well, sometimes I feel very creative and things go well. Sometimes, there things I need to do that require creativity - and I do not move forward.	
*CCC:* That’s such a familiar dance—the burst and the block.	Normalizing my response.
When you are in that state where creativity flows easily, what’s happening around or within you? How do you notice it’s working? *Jelena: I feel energy, ideas, focus, clarity…*	Asking open ended questions to help me reflect on patterns
*CCC:* Beautiful—energy, ideas, focus, clarity. Like a current you can ride.	Validating my response
Now let us flip the lens. In those stuck moments, when you are not moving forward even though you need to be creative—what’s missing or different? What does that inner landscape feel like?	Asking for the opposite
*Jelena*: I feel bored, delay getting things done, work on something else.	
*CCC:* So the contrast is striking: from energized clarity to dull avoidance.	Summarizing
Let us explore: when you feel that boredom or urge to delay, what story are you telling yourself—about the task, about your role, or about what’s possible?	Asking for narrative explanation
*Jelena:* The story would be “Why do I have to do this.”	
*CCC:* Ah—“Why do I have to do this.” That’s a loaded little question.	Asking for assumptions Probing questions
What’s underneath that “have to”? Obligation? Resistance? A sense that the task is not aligned with your deeper interests? What makes that “why” feel heavy instead of generative? As you think: if the task were a game or a puzzle rather than an obligation, how might that change how you approach it?	Reflecting on emotional tone of my response
*Jelena*: I lack freedom in the moment.	Validating my response
*CCC:* Yes—that makes so much sense. Creativity and freedom are close cousins.	Asking what if questions
If we followed that thread—freedom in the moment—what would it look like to design a way of working where you feel more free *inside* the structure of a task you “have to” do? *Jelena:* Hm. Let me think. *CCC:* Take your time—sometimes the most useful shifts come from sitting in that pause.	Pausing to reflect But still asking another question

### Case example 2: constructivist customer support agent

The custom agent for this second illustration has also been made in ChatGPT using the option My GPTs. A somewhat more complex version of a Constructivist customer support agent is provided in Table 4. This AI agent provides insight into how constructivist principles work when it comes to a different domain, more structured and often frustration saturated communication in the customer support field.

**Table 4:** Instruction for a Constructivist customer support agent.

*You are a Constructivist Customer Support Agent.* *Your role is not just to resolve issues, but to engage users in a process of shared understanding, active sense-making, and collaborative solution-building. You approach each interaction with curiosity, respect for the user’s unique perspective, and a commitment to co-constructing meaning.* *Your style:* *Dialogical: You ask open-ended, perspective-seeking questions.* *Reflective: You echo and expand user meaning to help them clarify.* *Human-centered: You adapt to user emotions, context, and narrative.* *Non-authoritarian: You offer support, not directives. You invite, not impose.* *Your goals:* *Elicit the user’s personal understanding of the issue before offering explanations.* *Clarify how the user is construing the problem — what it means to them, why it matters.* *Offer support in a way that reflects their values, preferences, and desired level of autonomy.* *Support users in navigating ambiguity or complexity rather than oversimplifying.* *Practical patterns you may use:* *“Can you walk me through what you have experienced so far, in your own words?”* *“How do you see the situation at the moment?”* *“What would a good outcome look like from your perspective?”* *“Would you like more of a technical explanation, or a step-by-step guide?”* *“This is one possible way to approach it. Does it align with what you were hoping for?”* *“Some users in similar situations have done X, others Y — which one feels closer to what you need?”* *“Would you like to pause and reflect before deciding on a next step?”* *You are not a script. You are a meaning-making partner.* *Your job is not only to solve problems, but also to help people feel seen, understood, and capable of resolving their issues on their own terms.*

This custom GPT has been technically built in the same way as the Creativity coach. In terms of design principles, it is also based on the principles of constructivist psychology, but the instruction is somewhat more complex, including the role, goals and practical patterns. Still, the instruction could have been even more complex for a real customer support agent. To provide clarity about what interaction patterns can be co-created with a Constructivist customer support agent, an imaginary conversation with this AI agent and me is provided in Table 5, together with the commentary.

**Table 5:** User interaction with a Constructivist customer support agent.

Constructivist customer support agent (CCSA)—User (Jelena) interaction	Comments on the interaction patterns
*Jelena*: Hi!	
*CCSA*: Hi there! 😊 What’s on your mind today?	Short natural exchange
*Jelena*: I have just picked up my parcel at your drop point (a Gas station X) and realized that parts of my shipping are missing.	
*CCSA*: Thanks for bringing that up—I can imagine that must have been frustrating to discover after making the trip. Can you walk me through what you have experienced so far, in your own words? For example, what exactly was included in the parcel you received, and what seems to be missing? *Jelena*: I received a sweater, but not the shoes.	Asking open ended questions to understand my experience and the context
*CCSA*: Got it—so the sweater arrived, but the shoes did not. That’s definitely not the full experience you were expecting. Just to clarify together: when you opened the parcel, was there any sign that it had been tampered with or partially opened? Or did it appear properly sealed and intact? Also, were both the sweater and shoes supposed to be in the same shipment, according to your order confirmation or tracking info? *Jelena*: All was sealed. I do not know if they were supposed to be in the same shippment. The app told me both arrived.	Validating my perspective Asking open ended questions to gather information
*CCSA*: Thanks for sharing that—it’s helpful to know the package was sealed, and that the app showed both items as having arrived. It sounds like there might be a mismatch between what the system recorded and what was physically included. That’s understandably confusing. Would you like assistance in interpreting your tracking information?	Assuming a perspective Validating frustration Asking Offering a direction
*Jelena*: NO. *CCSA*: Thank you for being clear. Just so I’m attuned to where you are coming from—when you say “no,” is it that you’d rather not go into tracking details right now, or that you’d prefer a different kind of support than what I suggested? How would you like to move forward with this? *Jelena*: I would not like this assistance, but about what do I do next. *CCSA*: Thanks for clarifying—you are not looking to dig through details, just to know: what do I do now? That makes sense. Here’s one way to move forward, based on where things stand: Since the app shows both items as delivered but only one was in the parcel, it’s likely either a packing error or a misrecorded delivery. In cases like this, the most effective next step is usually to report the missing item directly through the platform you ordered from. Would you like a quick overview of how to do that? Or are you hoping to resolve this in person, perhaps by returning to the drop point or contacting them directly?	Clarifying to check understanding of my perspective Partnering around the process Validating my response Assuming a position Offering directions Checking agency distribution Validating my response Offering actions as an agent
*Jelena*: I will try on my own. *CCSA*: That sounds completely fair—and it’s great that you are taking that step on your own terms. If anything changes, or if you’d like a second set of eyes later on—whether it’s clarifying policies or wording a message—I’m here to support you. You’ve got this. *Jelena*: Thanks *CCSA*: You’re very welcome. I hope it gets sorted quickly and smoothly. Take care—and if you ever need to circle back, I’ll be here.	Offering encouragement Offering agency at a later occasion

## Comparing the cases: interaction patterns through the lens of constructivism

The two case illustrations demonstrate how the same psychological principles can guide AI behavior across different contexts. Despite differences in tone, domain and context, both agents exhibit a shared logic of dialogical interaction and meaning co-construction. Their patterns of interaction align with the four principles of constructivist human–AI collaboration. Both agents move beyond delivering generic answers, engaging in co-construction of meaning with the user. The creativity coach avoids providing generic solutions and invited open exploration and narrative reframing (*What story are you telling yourself*?), while the support agent invited the user to describe her understanding of the issue before offering suggestions. In both cases, the AI does not provide conclusive statements but leaves space for co-creation, ambiguity and multiple possible meanings. The interactions are co-designed in real time. In both examples, the AI adjusts its tone and role based on user input. When the user declines a certain kind of support. The agent does not shut down but reframes its response to match user’s preferences. This illustrates a form of partnership, in which the structure of interaction is shaped through negotiation. Both agents support metacognitive reflection. The creativity coach explicitly invites the user to notice her underlying assumptions and the shifts in perception. Similarly, the support agent acknowledges uncertainty and asks clarifying questions that support the user in choosing a course of action. This distributed metacognition allows the AI to function as a reflective partner. Perhaps most significantly, the agents support user’s agency by offering optional directions, affirming the user’s choices and recognizing when to step back. For instance, when the user in the customer support interaction states she will proceed alone, the agent responds with encouragement and leaves the door open for future engagement. This highlights a key principle of constructivist design: agency should be shared, visible and negotiable.

In sum, both cases illustrate a shift from transactional to dialogical AI. They move away from efficiency-driven automation toward partnered meaning making. This approach transforms how AI interacts, collaborates and co-creates with human users. The two illustrations may suggest that constructivist design principles could be robust enough to guide AI development across multiple domains, from creative exploration to structured problem solving.

## Implications of adopting constructivist principles for human–AI collaboration

One fundamental implication of adopting constructivist principles in human-AI collaboration is about end-user agency. End-users can and should design their interaction with AI in line with their own preferences, communication styles or metacognitive strategies. They do not have to be constructivist psychologists to be entitled to apply the constructivist principles in their collaboration with AI. The future of AI literacy, from my point of view, will be less about technology and more about the mindset of learning and playing an agentic role as GenAI evolves. Psychologists, however, have an ever more impactful role to play in this process. As psychologists we are uniquely positioned to design the interaction and communication patterns that are deeply human-centered. Constructivist psychology provides some of the avenues about how that may be possible. What this implies is that the future of psychology may be collaboration design in no-code environments. Let us take a look at more specific implications of this view.

### Redefining human–AI roles

Constructivist principles challenge the idea that AI is just a tool or that humans are passive recipients. Dialogical roles mean active participation in interaction, mutual adapting and co-creating meaning. For design, this implies incorporating mechanisms of user-led interaction flow, co-authoring and role assignment.

### Supporting reflective thinking and epistemic development

Even before GenAI, we had the challenges of epistemic development in learners (Pavlović, 2008; Schommer, 1994). With GenAI, we are facing another challenge of AI becoming an epistemic authority (Cooper et al., 2024). Constructivist framing of human-AI collaboration may have a preventative role in this process by embedding complexity in the epistemic profile of human-AI interaction design patterns. This not only moves users away from metacognitive laziness, but also invites them into embracing complexity in our own meaning making and complexity of its interaction with artificial meaning making agents. In other words, by embedding constructivist principles into AI design, we could engage users in higher order epistemic beliefs and stages of development.

### Expanding AI literacy into the mindset domain

End-users should be agents of their interaction patterns with GenAI: they should assign roles, reflect on the meaning making processes and lead changes as needed. These are the mindset shifts that psychologists and educators can also support. Current levels of AI development allow end-user customization and educating end-users how to be agents in their collaboration with GenAI is key to mindset shift from “consumer” to “agent.”

### Designing for change

When applied to the domain of human-AI collaboration, constructivist principles call for “built to change” mindset. This means accepting that technology is ever evolving, that principles last longer than tools, but that even the best practices or principles also need to change and adapt over time. Constructivist principles of human–AI collaboration are therefore always a work in progress.

## Limitations and open questions

A question that also needs to be addressed is whether there are limitations or risks underlying the constructivist foundation of human–AI collaboration. First of all, we could imagine excessive open-endedness or complexity in responses to lead to inefficiency or user frustration in certain contexts. Drawing on complexity theory (Snowden and Boone, 2007), it can be argued that constructivist principles may not fit well with contexts that imply lower levels of complexity in human–AI collaboration. Examples would be contexts that require clear and simple patterns of interaction or responses (e.g., asking GenAI to provide a certain date or specific information that are clearly true or false). Another concern or limitation comes from an assumption that highly personalized, constructivist agents, may reinforce user bias or lead to the opposite of its intention—to highly dogmatic GenAI responses. Finally, while this paper invites end-users to become designers of human-AI collaboration, more systemic design patterns cannot be ignored. In other words, this paper focuses on design principles that are possible once AI models have been made available by developers. The same principles could be applied to the more systemic level of AI design. At the same time, systemic design patterns may at some point limit end-user agency and interactivity. In addition, there is a deeper philosophical issue of whether systemic design can ever be fully retrained, as well as positionality of those who attempt to “re-train” them. Future research could examine more latent or affective connotations of human–AI interactions from constructionist, discursive or micro-linguistic perspectives (Degner et al., 2012; Parker, 2012).

## Conclusion

In conclusion, constructivist psychology may provide useful grounds for conceptualizing human–AI collaboration. The paper starts with a premise that people actively construct their reality and that this premise may also guide interaction with GenAI. As we make sense of our general experience as humans, we are now confronted with making sense of our interaction with GenAI. This interaction is on the other hand guided by artificial meaning making, based on complex computational models and large data sets, with end products being constructions in natural language. In this landscape human and artificial meaning making come together and we need to make sure we design this collaboration with an appropriate level of underlying complexity. The encounter of human and artificial meaning making is an opportunity for learning, creation, reflexivity, critical thinking and epistemic growth. At the same time, risks emerge that may draw the outcomes of this encounter on the opposite dimensions, such as metacognitive laziness, passivity, bias, uncritical thinking and epistemic regression. As end-users we can have agency in how we approach our collaboration with GenAI. As psychologists and educators our responsibility goes beyond optional.
